# In Nature, There Is Only Diversity

**DOI:** 10.1128/mBio.02149-17

**Published:** 2018-01-02

**Authors:** Michael R. McLaren, Benjamin J. Callahan

**Affiliations:** a Department of Population Health & Pathobiology, North Carolina State University, Raleigh, North Carolina, USA; b Bioinformatics Research Center, North Carolina State University, Raleigh, North Carolina, USA

**Keywords:** metagenomics, microbial ecology, microbiome

## Abstract

Microbial ecology has been transformed by the advent of high-throughput marker gene and metagenomic sequencing methods. These tools provide expansive descriptions of microbial communities, but the descriptions are framed in terms of molecular objects, such as 97% ribosomal operational taxonomic units (OTUs), rather than biological objects, such as species. A recent study by A. B. Chase and colleagues (mBio 8:e01809-17, 2017, https://doi.org/10.1128/mBio.01809-17) explores the so-called microdiversity within the *Curtobacterium* OTU, the most abundant OTU in a leaf litter community. Perhaps unsurprisingly, they find that some important ecologic traits, such as drought response, are coherent within the OTU, but that others vary significantly. Here we discuss their findings in relation to the more general issue of how molecular tools can be effectively used to study microbial ecology. We specifically note the need for investigators to choose the right molecular methods for their biological problem, as nature does not respect the limitations and conventions associated with our methods.

## COMMENTARY

When we try to make sense of microbial communities, we often conceive of a cast of coherent species playing roles determined by their phenotypes and traits. However, the advent of high-throughput DNA sequencing has changed how we observe microbial communities in ways that can distance us from that conceptual framework.

Marker gene and metagenomic sequencing (MGS) methods provide expansive descriptions of microbial communities, but the descriptions are framed in terms of molecular measurements that are at best proxies for species and phenotypes. For example, microbial species (to the extent that they exist) are difficult to define and demarcate, while the MGS proxy for a species—the 97% identity ribosomal operational taxonomic unit (OTU)—is clearly defined and can be measured by low-cost marker gene sequencing. Our measurements of molecular objects are shaped by technical limitations, such as sequencing error, and by analytic choices, such as percent identity thresholds, that can begin as technical choices and mature into deeply rooted conventions. The accessibility of MGS methods has led to a growing prominence for molecular objects like OTUs in how we think and talk about microbial communities, thereby abstracting us one step further from biology and making it easy to forget these limitations and conventions. Without care, we can engross ourselves in the ecology of molecules rather than of microbes.

In a recent *mBio* article (1), Chase and colleagues investigate the prominent soil genus *Curtobacterium*. In previous work as part of the Loma Ridge Global Climate Experiment, the authors established *Curtobacterium* as the most abundant bacterial genus in Southern California leaf litter communities (2). The high abundance of bacteria relative to fungi (3) and the presence of genes in *Curtobacterium* isolates for decomposing complex carbohydrates found in leaf litter (4) further suggest a key role for this genus in the ecosystem.

Known *Curtobacterium* strains contain 16S rRNA genes that are at least 97% similar; thus, *Curtobacterium* strains have typically been grouped into a single object by previous OTU-based analyses. Chase and colleagues previously found significant genomic diversity within sequenced *Curtobacterium* isolates (4). In the present study (1), the authors explore the potential functional consequences of this so-called microdiversity within the *Curtobacterium* OTU. This work also contributes to a larger research program pursued by the authors and others investigating the phylogenetic scales over which various microbial phenotypes are conserved and remain coherent (reviewed in reference 5). Besides informing our understanding of ecologic diversification, this larger program is crucial for determining how to best use molecular methods to study microbial ecology.

In their study, Chase and colleagues (1) combined shotgun metagenomics, whole-genome sequencing, and laboratory measurements of cultured isolates to probe the genetic and phenotypic diversity within the *Curtobacterium* OTU. A comparison of the genomes of 16 isolates revealed significant variation in nucleotide sequence, protein sequence, and gene content and led the authors to demarcate six distinct *Curtobacterium* clades. Metagenomic sequencing data collected longitudinally across control, drought, and nitrogen enrichment treatments were mapped onto the isolate genomes, revealing the dynamics of each *Curtobacterium* clade. The ability of each isolate to degrade cellulose and xylan, the most abundant complex carbohydrates in leaf litter, was directly tested by laboratory growth assays at two different temperatures.

Some traits were coherent across *Curtobacterium* microbes, but others varied significantly. All clades of *Curtobacterium* increased their relative abundance in response to drought conditions, and all but one of the strains isolated was able to degrade xylan and cellulose at the hypothesized optimal temperature of 22°C. However, certain clades responded more strongly than others to drought conditions, and major variation between and within clades was observed in xylan and cellulose degradation at the hypothesized maximum viable temperature of 37°C.

These findings are consistent with a larger body of work showing that the phylogenetic depths over which ecologically relevant traits are conserved ranges widely among traits, from the deepest evolutionary branches to well below the species level (5). Tolerance to moisture variation in soil bacteria is conserved at the phylum level (6), while nitrogen fixation and carbon substrate preference are conserved only within species (5). Phylogenetic conservation can be exceptionally shallow when strong selection drives rapid adaptation of a trait, as observed for iron scavenging in ocean-dwelling *Vibrionaceae* (7) and virulence in a number of human-associated bacterial species (5).

This variation in phylogenetic conservation among traits has clear implications for marker gene and metagenomic studies. We would like to use molecular objects as easily measured proxies for microbial functions within a community, but for this to be successful, those objects must effectively track the relevant functional traits. This will not be the case if our molecular objects group together strains that differ in these traits. Therefore, the phylogenetic resolution of the molecular objects we ultimately analyze must be commensurate with, or finer than, the phylogenetic conservation in the relevant traits.

**FIG 1:**
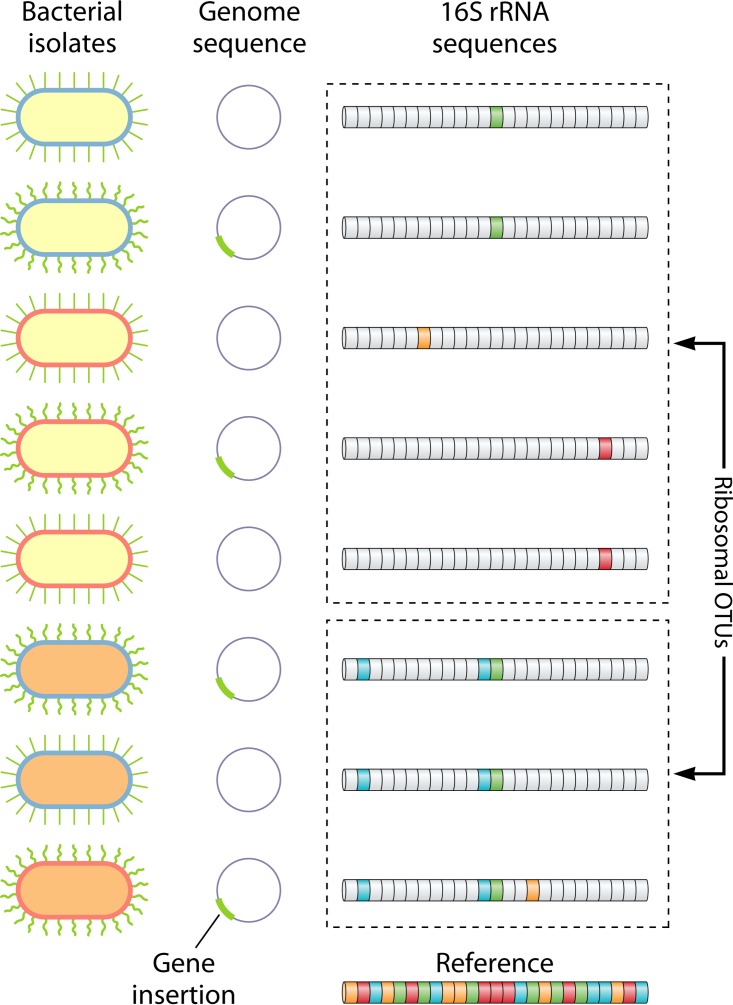
The phylogenetic depths over which different microbial traits are conserved varies widely, as does the resolution of different molecular analysis methods. In this hypothetical example, three cellular phenotypes—the shape of pili and the colors of the cell wall and the cell interior—exhibit different scales of phylogenetic conservation. Variation in pilus shape evolves rapidly by gene transfer and can be tracked by whole-genome sequencing methods but not by 16S rRNA sequencing. Cell wall color evolves consistently with the organismal phylogeny and thus can be tracked by exact 16S sequences, but changes too rapidly to be coherent within ribosomal operational taxonomic units (OTUs). Only the slower evolving cell interior color is effectively tracked by ribosomal OTUs. It is important that investigators carefully consider the resolution of the molecular methods they employ and whether that resolution is sufficient to track the traits that are relevant to their study system and scientific question.

Because different traits are conserved at different levels, the most appropriate molecular method and analysis will depend on the system being studied and the question being asked (Fig. 1). In this study (1), the drought response of *Curtobacterium* was coherent across the *Curtobacterium* OTU, and thus, amplicon sequencing of the 16S rRNA gene could be an effective tool for studying microbial drought response in leaf litter. However, the substantial differences in cellulose and xylan degradation capacity at high temperatures suggest that a different method that can resolve different *Curtobacterium* strains may be more appropriate if temperature extremes are expected to be an important factor.

As genetic data are only ever a proxy for phenotype, the need to match the resolution of molecular methods to the scale of variation in functional traits applies not just to marker gene methods but to all analyses based on DNA sequencing. The analysis of shotgun metagenomic data also relies on explicit or implicit similarity thresholds within which diversity is effectively ignored. When mapping reads to a set of references, for taxonomic assignment or otherwise, the incompleteness of any reference database necessarily leads to some genetically and phenotypically distinct sequences being mapped to the same reference. Similarly, the genomic bins used in the creation of metagenome-assembled genomes can lump together distinct strains that are too similar for the assembly or binning algorithms to distinguish.

Molecular approaches that treat the gene, rather than the species, as the fundamental biological object also depend on similarity thresholds that can group together phenotypic diversity. Shotgun metagenomic data are commonly mapped against databases of microbial genes to obtain presence/absence and relative abundance measurements for orthologous genes. Gene-level approaches sidestep the disconnect between functional diversity and organismal phylogeny that can be caused by processes like horizontal gene transfer, but orthologous gene clusters are still aggregates of similar but different genes and thus can group together relevant phenotypic variation. For example, the ability to grow on a particular carbohydrate source might be determined by the presence of an orthologous gene cluster defined at a 10% divergence level, while the growth rate at the prevailing temperature in the environment studied may vary substantially between different orthologs in the cluster.

Too often, molecular and bioinformatic methods are chosen mainly for their ease or popularity. The tremendous variation in the phylogenetic scales over which different phenotypes are conserved requires more attention to matching the molecular objects we use in our analyses with the microbial ecology we wish to understand. This issue is perhaps most widely recognized for amplicon sequencing of the 16S rRNA gene, which is understood to be effective for some problems, such as diagnosing dysbiosis in the vaginal microbiome (8), but is known to aggregate variation too broadly for other problems such as identifying Shiga toxin-producing *Escherichia coli*. However, the problem of matching molecular objects with the scale of the relevant phenotypic variation is not a challenge for amplicon sequencing alone; it is a challenge for all molecular methods used to study patterns and processes driven by function.

This study by Chase and colleagues (1) represents the latest in the long, hard, taxon- and trait-specific work of relating genetic differences to functional phenotypic variation. Their investigation of functional diversity within the *Curtobacterium* OTU revealed that some ecologically relevant traits were coherent across the OTU, while others varied within the OTU. This should not be surprising. There are no 97% OTUs in nature. There is no “microdiversity” in nature. There is only diversity. Nature does not respect the limitations and conventions associated with our methods; therefore, choosing the right tools for a given study may require a break from convention, and careful consideration of the match between potential molecular methods and the functional variation important to our scientific problem.
